# CMR-derived aortic stiffness is associated with more rapid aortic dilation and surgical replacement in children and young adults with connective tissue disorders

**DOI:** 10.1186/1532-429X-16-S1-O48

**Published:** 2014-01-16

**Authors:** Ashwin Prakash, Himanshu Adlakha, Cara Hass, Shaine A Morris, Tal Geva, Ronald V Lacro

**Affiliations:** 1 Cardiology, Boston Children's Hospital, Boston, Massachusetts, USA; 2 Section of Cardiology, Department of Pediatrics, Baylor College of Medicine, Houston, Texas, USA

## Background

Aortic stiffness measures have been proposed as novel indicators of disease severity in patients with connective tissue disorders (CTD). However, these measures have not been studied extensively in children. Their relationship to adverse aortic outcomes in this population is also unknown. We studied CMR parameters of aortic stiffness in children and young adults with CTD and assessed their relationship with the rates of aortic dilation and surgical root replacement.

## Methods

Existing CMR studies on CTD patients in the period 2005-2013 were retrospectively reviewed. Patients with aortic root replacement prior to CMR were excluded. Aortic strain [(systolic area-diastolic area)/diastolic area] and distensibility (strain/brachial pulse pressure) were calculated using direct planimetry on short axis SSFP images of the aortic root, ascending aorta (AAO) and descending aorta (DAO). AAO distensibility data were compared to published normative data. Aortic root distensibility values were compared with published normal values in a similar age group.

## Results

A total of 83 patients with CTD were included (Marfan syndrome 45, Loeys-Dietz syndrome 10, Ehlers Danlos syndrome 1, non-specific CTD 27); median age 24 years (range 1-55); 17% under age 18 years; 60% male. Compared with published controls, aortic distensibility was lower in CTD patients in the aortic root (2 ± 1.2 vs. 9.1 ± 4.7 × 10-3 mm Hg-1, p < 0.0001). Median z-score for AAO distensibility calculated from published normative data was -1.93 (range -8.7 to 1.3). Stiffness measures did not differ significantly between CTD subtypes. For each patient, strain and distensibility values in the root, AAO and DAO were significantly correlated. Aortic strain and distensibility decreased with increasing patient age. Patients with larger aortic roots demonstrated lower root strain (p = 0.001) and root distensibility (p = 0.05). In follow up, no aortic dissections occurred but 16/83 patients underwent aortic root replacement. As dictated by current treatment guidelines, aortic root dimension and area were the strongest predictors of surgery. However, lower aortic root strain was also predictive of surgery even when controlling for patient age (p = 0.02, odds ratio 1.2; 95% CI 1.02-1.38 for every unit decrease in percent systolic strain). Patients with higher AAO strain (controlling for age) showed more rapid increase in echocardiographic aortic root diameter z scores over a mean follow up period of 6 years (p = 0.02).

## Conclusions

Patients with CTD demonstrate increased stiffness of all aortic segments. Patients with the largest aortic root dimensions have the stiffest aortas. Higher aortic stiffness is associated with more rapid aortic root dilation and higher likelihood of aortic root replacement. In order to refine current treatment guidelines that rely on aortic size alone, further studies should examine whether parameters of aortic stiffness are independently predictive of dissection and death in patients with CTD.

## Funding

None.

